# Empirical exploration of whale optimisation algorithm for heart disease prediction

**DOI:** 10.1038/s41598-024-54990-1

**Published:** 2024-02-24

**Authors:** Stephen Akatore Atimbire, Justice Kwame Appati, Ebenezer Owusu

**Affiliations:** Department of Computer Science, University of Ghana, Accra, Ghana

**Keywords:** Heart disease, Whale optimization, Feature selection, Accuracy, Swarm intelligence, Mathematics and computing, Applied mathematics, Computational science, Computer science, Cardiovascular diseases

## Abstract

Heart Diseases have the highest mortality worldwide, necessitating precise predictive models for early risk assessment. Much existing research has focused on improving model accuracy with single datasets, often neglecting the need for comprehensive evaluation metrics and utilization of different datasets in the same domain (heart disease). This research introduces a heart disease risk prediction approach by harnessing the whale optimization algorithm (WOA) for feature selection and implementing a comprehensive evaluation framework. The study leverages five distinct datasets, including the combined dataset comprising the Cleveland, Long Beach VA, Switzerland, and Hungarian heart disease datasets. The others are the Z-AlizadehSani, Framingham, South African, and Cleveland heart datasets. The WOA-guided feature selection identifies optimal features, subsequently integrated into ten classification models. Comprehensive model evaluation reveals significant improvements across critical performance metrics, including accuracy, precision, recall, F1 score, and the area under the receiver operating characteristic curve. These enhancements consistently outperform state-of-the-art methods using the same dataset, validating the effectiveness of our methodology. The comprehensive evaluation framework provides a robust assessment of the model’s adaptability, underscoring the WOA’s effectiveness in identifying optimal features in multiple datasets in the same domain.

## Introduction

Heart Disease (HD) is of utmost importance due to the heart’s critical role among other human organs. HD has high death rates worldwide, with approximately 17.9 million people dying from heart conditions in 2019^1^. Heart diseases account for 32% of global deaths, with heart attacks and stroke alone making more than 85% of recorded deaths. Over 75% of cardiovascular deaths in 2019 occurred in underdeveloped nations, accounting for 38% of deaths under 70 years^1^. Since cardiovascular diseases are fatal, their early detection will enable medical professionals to provide timely healthcare to patients to avert death.

Because of a scarcity of ultra-modern examination tools and medical experts, conventional medical methods for diagnosing heart diseases are challenging, complicated, time-consuming, and exorbitant, making the diagnosis of heart diseases difficult and sometimes unavailable, especially in developing countries^2^. Machine and deep learning methods have been recently used to analyze clinical data and make predictions^3^.

Machine learning (ML) provides cost-efficient alternatives where already collected patient data serve as a data mine to perform predictive analysis for diagnostic purposes. To improve the accuracy of ML models, some existing works have focused on using various classifiers or their enhanced forms^4–7^. Related works confirm that the feature selection reduces data dimensionality and improves model performance significantly^8^. Hence, some studies have utilized various methods to improve performance by varying the feature selection methods^9,10^.

However, some works that utilize feature selection are fraught with redundant features that impact metrics recorded. This is affirmed when wrapper methods are used over filter methods and when embedded methods are used over filter and wrapper methods. It also explains why works, including feature selection, may only record better performance on some datasets if the technique is efficient. In addition, though the researchers do not present the reason some existing works have not reported on specific metrics, studies such as Hicks et al.^11^ have posited that in a clinical setting, a subset of metrics may give an erroneous outlook of how a model performs and not enabling holistic model performance evaluation. There is an avenue for more scientific work on feature selection methods capable of improving other metrics besides the accuracy metric. This helps to affirm the reliability of the model performance as the unavailability of multiple evaluation metrics is an indication of an unbalanced model not capable of being thoroughly assessed.

This study proposes the use of the whale optimization algorithm (WOA) as a swarm-inspired feature selection algorithm on five (5) heart datasets on ten (10) models (classical ML, ensemble and deep learning models) for the selection of relevant datasets features. The approach contributes to the body of knowledge in the heart disease domain by providing a comprehensive assessment of five different datasets (in the same domain), ten different models and five evaluation metrics. The proposed methodology also validates the robustness of the WOA algorithm on five datasets of variable sizes in the same domain compared to most works, which do not test their methodologies on multiple datasets in the same domain.

## Related works

### Introduction

Identifying significant features (or redundant features) in a dataset remains a critical activity in modelling^12^. Feature selection helps to determine relevant subsets from the existing features^13^ and is recommended for use where one needs to understand the selected features^14^. Excessive dataset features cause over-fitting, reduce model efficiency, and impair model generalization^15^. Feature selection (FS) improves the modelling process^16^. Selected feature selection techniques in literature are mostly grouped into filter, wrapper, hybrid, embedded, and recently, swarm intelligent methods.

#### Filter-based feature selection methods

Filter-based methods do not consider the dependence of features on one another; only the intrinsic properties are considered^17^. Ghosh et al.^18^ used the Cleveland heart dataset alongside the Decision Tree, K-Nearest Neighbour (KNN), and Random Forest (RF) classifiers, recording the highest accuracy of 93.36% with RF. The most optimal features were selected using relief before being passed to the classifiers for training and testing. Relief selected six of the 13 features, resulting in a significant achievement of accuracy. Narsimhulu et al.^19^ proposed a Filter Based Feature Selection (FBFS) to detect relevant features and remove the redundant ones. RF recorded the highest accuracy of 95.08%, validating the positive influence of the filter feature selection method on model accuracy. Filter methods can compute the scoring function quickly and efficiently^20^. Filter methods do not consider feature interdependence and are not dependent on the classifiers^21^.

#### Wrapper based feature selection methods

To resolve some of the shortcomings of filter-based methods, the wrapper methods use classifiers and consider feature interdependence, increasing computational time^22^. Evaluating different feature subsets requires retraining and testing^23^ compared to the filter feature selection algorithms. El-Sayed employed the Genetic Algorithm (GA) wrapper method on the Cleveland heart disease dataset, obtaining 89.07% and 67.22% for binary and multiclass, respectively. First, GA was used to reduce the attributes from the dataset, and Linear Discriminant Analysis (LDA) was used for classification. MultiLDA and multi-classifiers were then used again after GA for multiclass datasets. In their study, GA used alongside the LDA classifier outperformed KNN, Support Vector Machine (SVM) and Naïve Bayes (NB). Sequential Backward Selection (SBS) is also applied to ascertain more significant features, increasing classifier accuracy while decreasing computational time in the study of Haq et al.^10^. The Cleveland heart disease database was used in the study, recording an accuracy of 90% with six features selected using SBS for feature selection. The KNN classifier with the SBS method outperforms the KNN classifier used single-handedly. The Recursive Feature Elimination (RFE), another wrapper method, is used in the study of^24^. Feature selection is performed using the SVM-RFE before using KNN to find the best features and reduce computing time. KNN without FS had an accuracy of 82.65% and 86.33% with feature selection. The Weighted KNN without FS recorded 83.45% and 90.88% with feature selection. The results confirm that with feature selection, the accuracy of the KNN or the weighted KNN experiences a significant improvement. Although wrapper methods perform better than filter methods, they are computationally expensive^23^.

#### Embedded based feature selection methods

Embedded methods search for classifier-specific optimal features and keep track of feature interdependence with less complexity than the wrapper method^25,26^. Embedded methods are less prone to overfitting than wrapper methods and are computationally costlier than the filter method^27^. For the prediction of HD, the LASSO feature selection was compared to the relief feature selection method by Ghosh et al.^28^ by using the the Cleveland, Long Beach VA, Switzerland, Hungarian and Statlog datasets. The study achieved the best accuracy of 99.05% with LASSO and the Random Forest Bagging classifier. In a related study, Zhang et al.^23^ proposed the LinearSVC algorithm, an embedded method with a Deep Neural Network using the Cleveland UCI dataset. The work resulted in an accuracy of 98.56%. The study confirms the advantage of embedded methods of feature selection over the former techniques. The embedded feature approach mitigates the shortcomings of the filter and wrapper methods by engaging with the classifier and accounting for feature dependencies^26^. Its drawback, however, is its slow performance^29^.

#### Hybird based feature selection methods

The hybrid methods use two or more methods together for feature selection tasks. The study of^30^ hybridized Cuckoo Search with the rough set to form the Cuckoo Search with the Rough Set (CSRS) model in their paper. Data from 603 patients are divided into train data for 332 and test data for 271. Cuckoo Search is applied to determine the most optimal features from the training data. Eight features with highly significant values are chosen after 1000 iterations. With optimal features selected, CSRS obtained 93.7% accuracy, outperforming other Cuckoo Search integrated models by 3.37%. Also, a three-phase feature selection approach is proposed by^31^. In their paper, a three-way feature selection method is proposed for reducing the feature set of the arrhythmia dataset. The three cancer datasets achieved 100% accuracy, and 94.50% was achieved on the arrhythmia dataset. The method selects features with the best accuracy not dependent on the filter methods and the classifiers in the first phase, fusing four techniques: Mutual Information (MI), ReliefF (RFF), Chi-Square (CS), and Xvariance (XV) using three classifiers, KNN, SVM, and NB. The original data set is then subjected to the XGBoost | algorithm, with the top features chosen based on accuracy. In the second phase, the top features in the previous stage are correlated using the Pearson Correlation Coefficient (PCC), and strongly correlated features are dropped, ensuring that the feature subsets have maximum relevance. Again, XGBoost is used to obtain the top features, which are then sent to Phase 3. The optimal feature set is finalized in the third phase, using WOA. Arrhythmia, leukaemia and two others form part of the four datasets used in the trials. The features with the most negligible significance in the dataset had the lowest rankings in information, reliance, and distance. It is also observed that the selected features were reduced by 150 and 1286 times for the arrhythmia and leukemia datasets, respectively. The proposed method enhances the removal of noisy data which are discarded and improves accuracy.

Aside from the authors’ report of limited data, one of the tri-stage’s flaws is the costly computation required to obtain accuracy during the first phase and when WOA is applied.

Arroyo and Delima^32^ propose a genetically optimized neural network for HD risk prediction. The Cardiovascular disease dataset with 70,000 records and 12 features was employed for ANN modelling. The authors’ work resulted in higher accuracy. However, determining the correct number of layers and neurons takes time and effort. The study of^33^ also presented techniques based on the KNN, SVM, NB, RF, and a Multilayer Perceptron (MLP) optimized by Particle Swarm Optimization (PSO) merged with Ant Colony Optimization (ACO). The study achieved a maximum of 99.65% accuracy, and the research further enhances the position that with an optimal result set, the accuracy of a study can be significantly improved^34^. Proposed a hybrid feature selection through Information Gain, Correlation, Chi-Squared, and Relief-F. Using the KNN classifier on a heart failure dataset. The approach recorded 84.61% accuracy. The computational and efficiency challenges inherent in a particular algorithm can be incorporated and reflected in the hybrid algorithm, making the hybrid method very complicated to implement. Therefore, individual algorithms’ challenges must be intentionally handled to improve efficiency and accuracy in hybrid methods.

#### Swarm intelligent based feature selection methods

Swarm intelligence optimization methods have emerged as robust methods for feature selection in many fields. “Swarm Intelligence (SI) is a type of artificial intelligence that is based on collective behaviours in decentralized and self-organized systems”^35^. SI is usually inspired by an organized pattern of a random behaviour of a population known as the agents. The intelligent behaviour results in a recognizable pattern helpful in solving optimization problems. Some standard swarm algorithms include the Cuckoo Optimization Algorithm (COA), Ant Colony Optimization (ACO), Bat Algorithm (BA), Grey Wolf Optimization (GWO), Salp Swarm Algorithm (SSA), Marine Predator (MP) and whale optimization algorithm (WOA).

Usman et al.^36^ propose the Cuckoo Search Algorithm (CSA) and another variant, the Cuckoo Optimization Algorithm (COA) for FS on the Eric, Hungarian, Stat log, and Z-Alizadeh datasets. Four classifiers are used in their study, namely Support Vector Machine (SVM), Multi-Layer Perceptron (MLP), Naïve Bayes (NB), and Random Forest (RF) classifiers. CSA outperformed COA by selecting fewer features and higher accuracy across all datasets. Other researchers, Al-Tashi et al.^37^, proposed establishing optimal features to diagnose coronary artery disease using the Grey Wolf Optimization (GWO). The authors first use GWO to determine the significant features. The SVM is then utilized with the optimally selected features as input. The Cleveland UCI dataset was employed, and the model recorded 89.83% accuracy. The other evaluation metrics used are sensitivity, which was recorded at 93%, and 91% for specificity rates. In the study of Al-Tashi et al.^37^, Grey Wolf Optimization (GWO) finds optimal features in the dataset and then evaluates the fitness function of GWO using SVM. The Cleveland UCI dataset used with the study recorded 89.83%, 93% and 91% in accuracy, sensitivity, and specificity, respectively. By combining GWO and the Naïve Bayes (NB) classifier, a new strategy for detecting cardiac disease is developed^38^. The features of the heart disease dataset are discretized to increase the accuracy of the classifiers. GWO then automatically selects Naïve Bayes’s weights to maximize NB’s performance, achieving 87.45% accuracy with good values for Sensitivity, F-measure, and G-mean. The Cleveland UCI data was used in the project. The proposed (GWO-NB) method performed better than the standard Naïve Bayes classifier in accuracy from the experiments. It also confirms the effectiveness of GWO on classifiers. To improve the GWO, the paper of Chakraborty et al.^39^ proposed an enhanced form of GWO for feature selection. Cleveland, Long Beach VA, and Switzerland were utilized in their study. Others include the Hungarian and Statlog datasets. Bagging and boosting techniques produce hybrid classifiers with NB, RF, Decision Tree (DT), K-Nearest Neighbor (KNN), Neural Network, Gradient Boosting, and Adaptive Boosting (AdaBoost). RF achieved the best accuracy of 99.26% with Enhanced-GWO, an improvement of accuracy of 11.90% over the conventional model. The Whale Swarm Algorithm (WSA) proposed by David^40^ was used to select significant features to determine the presence of cardiovascular disease. Using the Statlog dataset, the study recorded an average selection of 6 features after 100 iterations. Using the Logistic Regression (LR), Random Forest, Support Vector Machine and Gaussian Naive Bayes (GNB) classifiers, the study noticed that the Random Forest outperformed the other classifiers, reporting an accuracy of 85.7%. Another commonly used swarm intelligence algorithm within the heart disease domain is Particle Swarm Optimization (PSO). Shahid and Singh^41^ in their paper propose a model known as the emotional neural networks (EmNNs) that PSO has hybridized on the Z-Alizadeh Sani dataset. The researchers apply four distinct feature selection algorithms to optimize the performance of the suggested model. The proposed method selected a total of 22 features. The model outperforms all other models used in the study, recording the highest averages for accuracy, precision, sensitivity, specificity, and F1 score at cross-validation, i.e., 88.34%, 92.37%, 91.85%, 78.98%, and 92.12%, respectively, which are comparable to existing methods in the literature. Their work is improved by introducing a novel multi-objective PSO (MOPSO) proposed by Asadi et al.^42^. Using the Statlog, Cleveland, SPECT, SPECTF, VA Long Beach, and Eric datasets, the study recorded improved accuracy better than when feature selection was not used. Wankhede et al.^43^ propose a Decision Function-based Chaotic Salp Swarm (DFCSS) method to determine the most significant features after data pre-processing. The relevant attributes are then provided to an enhanced Elman neural network classifier. The experiment demonstrates that the proposed method outperformed existing methods with 98.7% and 98.0% accuracy for CVD and UCI datasets, respectively. The Salp Swarm Algorithm selects valuable features from the UCI Cleveland and heart-failure-clinical-records datasets in Sureja et al.^44^. The study recorded 98.75% and 98.46% accuracy for the respective datasets. Swarm-based feature selection approaches thus have the potential to enhance classifier performance drastically.

This research explored the use of WOA for feature selection for the following reasons. First, WOA is easy to implement due to the few internal numbers of parameters^45^, outperforms other swarm algorithms such as the Particle Swarm Optimization (PSO) and gravitational search algorithm (GSA) as recorded in the study of Mirjalili and Lewis^46^ when used for engineering design problems. It has provided outstanding results for optimization problems in domains such as wireless resource allocation and gold price forecasting (outperforming PSO, Grey Wolf Optimization and genetic algorithm^47,48^. The study of Ay et al.^49^ also affirms the creditable performance of WOA compared with cuckoo search (CS), flower pollination algorithm (FPA), and Harris Hawks Optimization (HHO) algorithms, other metaheuristic algorithms. Also, WOA is not widely explored in the heart disease domain; hence, its creditable performance in different fields, simplicity, and excellent output against other metaheuristic methods make it a choice worth selecting for FS in the HD domain that guarantees good results.

## Proposed methodology

**Figure 1:**
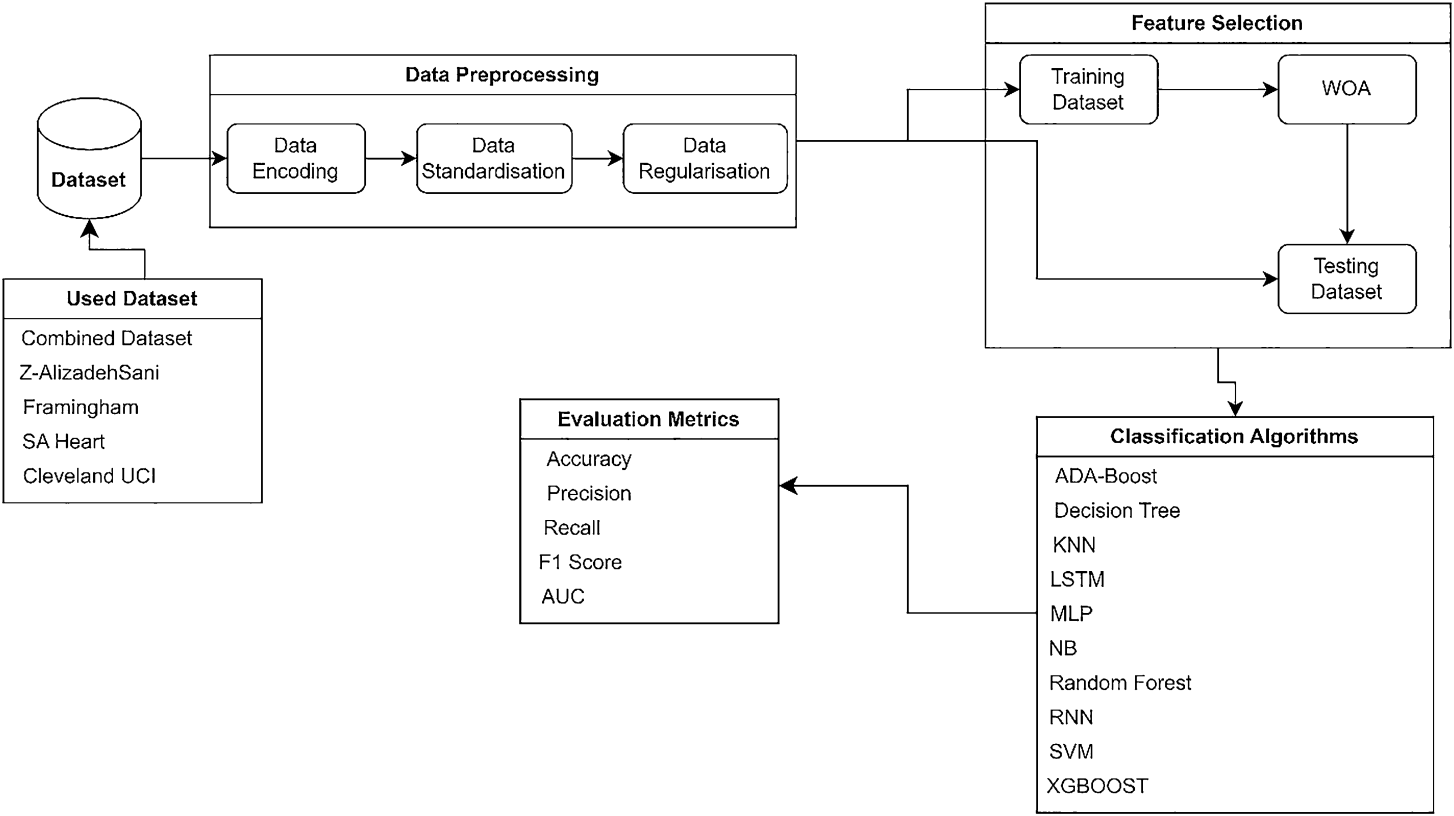
Methodology for the study.

The methodology for this work and the steps used for feature selection, model training, and model evaluation are discussed. Figure 1 depicts the proposed methods employed for this work.

### Datasets

The datasets leveraged in this study are summarized in Table 1. The activities performed on the datasets are discussed in the data pre-processing subsection following.

**Table 1:** Datasets utilized in the study.

#	Name of dataset	No. of observations	No. of features	Publicly available?
1	Combined dataset	1025	13	Yes
2	Z-AlizadehSani dataset	303	54	Yes
3	Framingham dataset	4133	15	Yes
4	South African heart dataset	462	9	Yes
5	Cleveland UCI heart dataset	303	13	Yes

#### Dataset visualization before feature selection

For each of the five datasets, four visualization techniques were employed to gain insights into the data’s structure prior to feature selection.

Density-Based Spatial Clustering of Applications with Noise (DBSCAN) is utilized to identify and visualize outliers, to aids the understanding of the data’s distribution and identifying anomalous observations. Correlation Heatmaps was utilized to examine the relationships between features while.

Boxplots was used for to visualize feature distribution. Histograms and density plots give insight on the target variable’s distribution across different classes or values.

**Figure 2:**
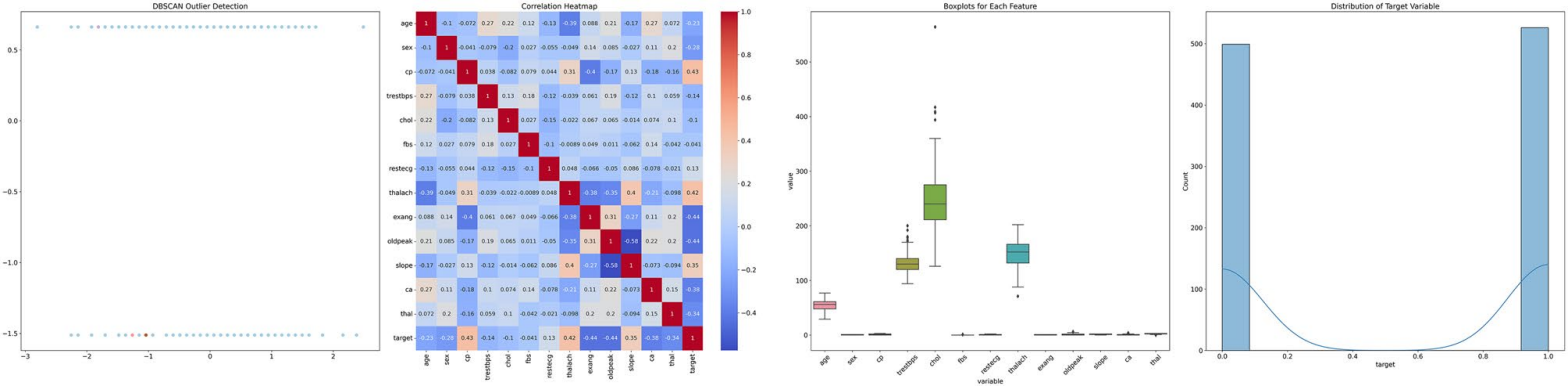
Combined dataset visualization before feature selection.

**Figure 3:**
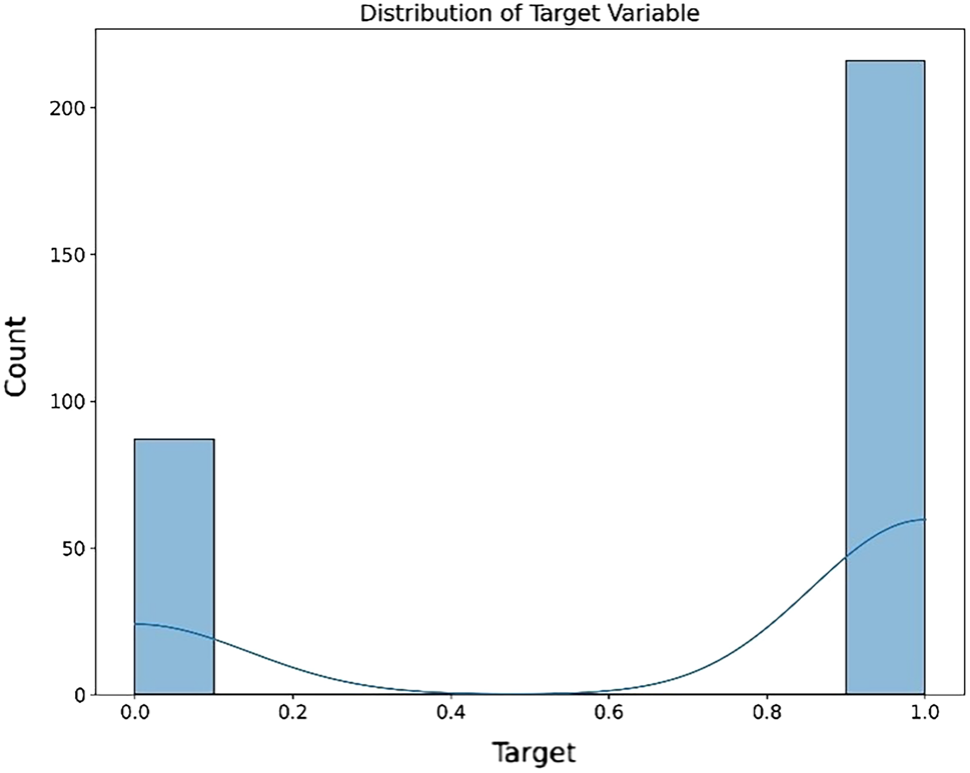
Z-AlizadehSani dataset target visualization before feature selection.

**Figure 4:**
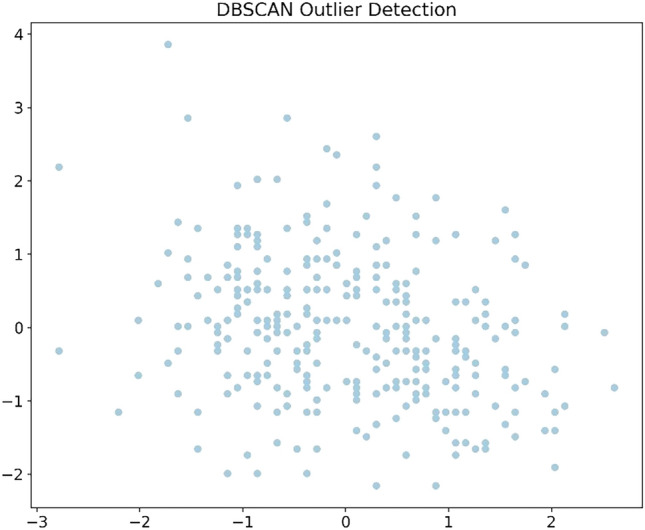
Z-AlizadehSani dataset DBSCAN outlier detection visualization before feature selection.

**Figure 5:**
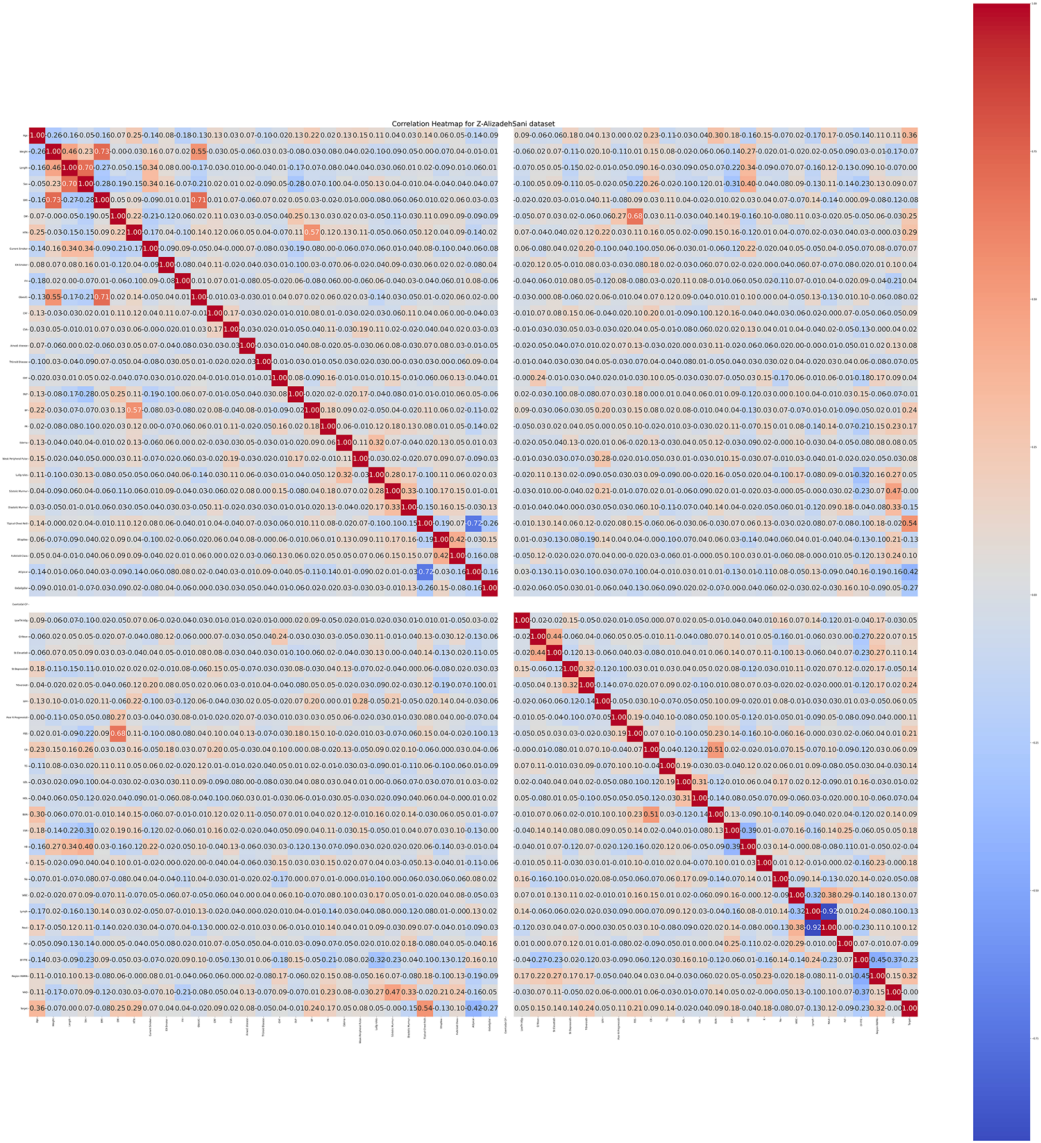
Z-AlizadehSani dataset correlation Heatmap visualization before feature selection.

**Figure 6:**
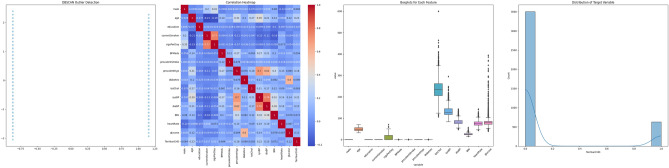
Framingham dataset visualization before feature selection.

**Figure 7:**
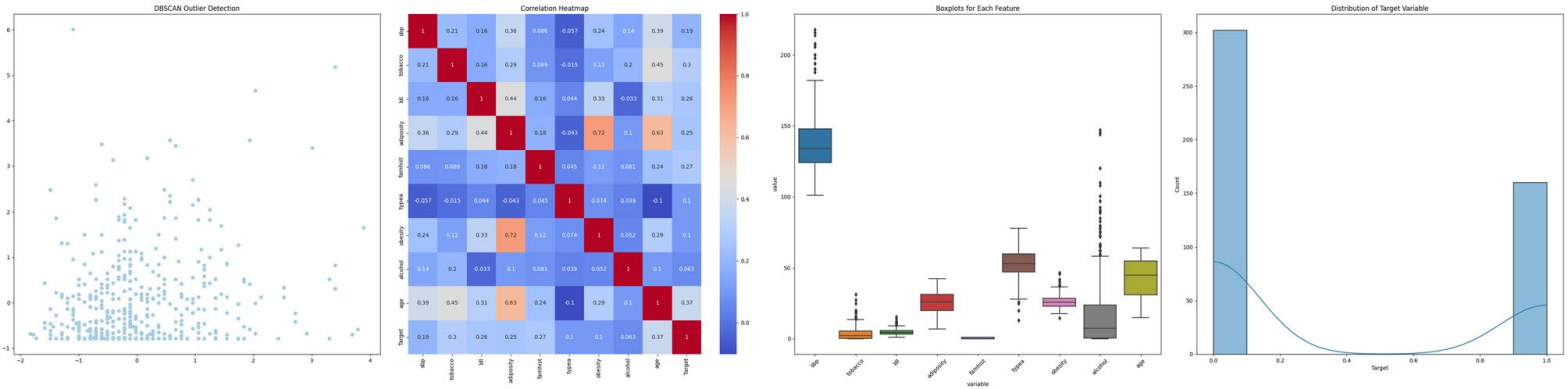
South African heart dataset visualization before feature selection.

**Figure 8:**
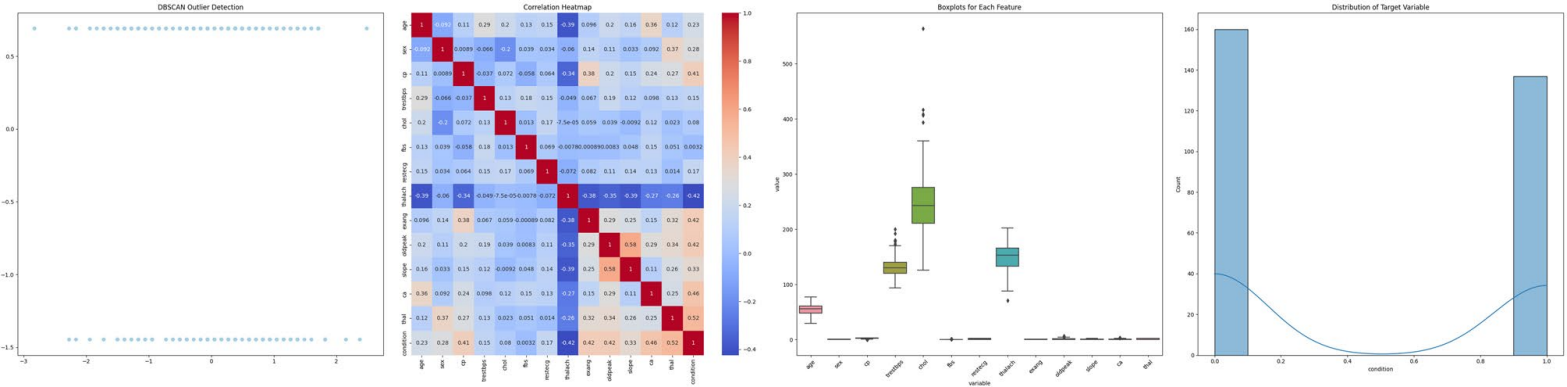
Cleveland UCI dataset visualization before feature selection.

The dataset visualizations reveal in the DBSCAN plot a lack of clear, separable clusters, or it could be indicating a need to adjust the algorithm’s parameters for a more meaningful clustering. The correlation heatmap provides insights into potential multicollinearity, which could influence feature selection and model performance. The boxplots on the other hand reveal the presence of outliers and the spread of the data. The target distribution plot shows a fairly balanced dataset. A general observation on the datasets reveal similar observations underpinning the need for the preprocessing tasks and feature selection. The visualizations for the datasets are shown in Figs. 2, 3, 4, 5, 6, 7 and 8 representing the combined, Z-AlizadehSani, Framingham, South African and the Cleveland UCI datasets respectively.

### Data pre-processing

Data pre-processing is an essential activity in most ML pipelines. It includes tasks such as data cleaning, transformation, and organizing data before passing it to a model. This step is essential to improve data quality and make it suitable for building accurate and efficient models.

In this work, the target values indicating the existence of HD are coded as 0 or 1 (absent and present, respectively). Other categorical data fields, such as “famhist” in the SA heart dataset with values “absent” and “present”, are coded as 0 for absent and 1 for present. “Male” and “Female” values for all datasets are encoded as 1 and 0, respectively. All other fields with similar values are coded with numerical values.

#### Data standardization and regularization

The standardization process utilizing the StandardScaler was employed. This involved adjusting the features present in the data to be transformed to possess a zero (0) mean and a one (1) standard deviation. Standardizing the features with StandardScaler has a lower error than MinMaxScaler because StandardScaler scales each feature with zero mean and unit variance, scaling them in an equivalent bell curve and handles missing values preventing overfitting^50,51^. A Logistic Regression model with an L2 regularization penalty is fitted with the standardized data. Another parameter, “C”, which controls the trade-off between fitting the data well and the simplicity (parsimonious) of the model, is utilized in the logistic regression model. The Logistic Regression classifier fits the model to the scaled train data and the corresponding target. The model’s coefficients, which indicate each feature’s significance in making predictions, are stored in a variable applied to the training data to obtain a new, regularized training dataset. This regularized training data is utilized for model training. Afterwards, the stored coefficient is applied to the test data to regularize the test data and target. Logistic regression with L2 regularization has proven useful in prior studies to promote sparse solutions^52,53^. The regularized train data is passed to the whale optimization algorithm to select the optimal features for model training modelling and testing. The WOA algorithm is discussed in the adjoining pages.

### The whale optimization algorithm (WOA)

WOA is an optimization algorithm that draws inspiration from nature and imitates humpback whales’ hunting techniques developed by Seyedali Mirjalili^46^. The main components of whale optimization are position updating, encircling prey, and searching for prey. The entire WOA steps are discussed in this section and modified to be utilized for feature selection, which is further explained.

#### Encircling prey

Encircling uses the first current solution as the target or near to it since the optimal design’s location is initially unknown. The remaining agents then update their locations guided by the location of the best agent. This behaviour is modelled by Eqs. (1) and (2).

1 $${\vec{\text{D}}} = \left| {{\vec{\text{C}}} \cdot {\vec{\text{X}}}^{*} ({\text{t}}) - {\vec{\text{X}}}({\text{t}})} \right|$$

2 $${\vec{\text{X}}}\left( {{\text{t}} + 1} \right) = {\vec{\text{X}}}^{*} ({\text{t}}) - {\vec{\text{A}}} \cdot {\vec{\text{D}}}$$

where $t$ is the current iteration, the coefficients ${\vec{\text{A}}}$ and $\vec{C}$ being vectors and ${\text{X}}^{*}$ being the absolute position vector value for the best solution of the iteration. ${\vec{\text{A}}}$ and $\vec{C}$ are determined using Eqs. (3) and (4).

3 $${\vec{\text{A}}} = 2{\vec{\text{a}}} \cdot {\vec{\text{r}}} - {\vec{\text{a}}}$$

4 $${\vec{\text{C}}} = 2 \cdot {\vec{\text{r}}}$$

where $\overrightarrow{a}$ linearly reduces from 2 to 0 and $\overrightarrow{r}$*,* a randomly generated vector with a value within $\mathrm{0,1}$ is generated.

#### Bubble-net attacking method (exploitation phase)

Bubble-net attack (exploitation) uses encircling and spiral updating techniques to control the whale mechanisms in WOA. The shrinking encircling mechanism is governed by Eqs. (2) and (3), with the varying range of vector A relying on vector a. Equation (3) can compute this range. The vector $a$ diminishes from 2 to 0 across numerous rounds, affecting vector A. When vector A lies within [− 1, 1], the agent’s following location is between the present and target positions. The spiral-based position update procedure begins by computing distances between the whale’s present position and the positions of the targets. Based on this range, the spiral motion mimicking the humpback whale’s swimming pattern is generated. The modelled pattern is explained in Eq. (5).

5 $${\vec{\text{X}}}({\text{t}} + 1) = \overrightarrow {{{\text{D}}^{\prime } }} \cdot {\text{e}}^{{{\text{bl}}}} \cdot \cos (2\uppi {\text{l}}) + {\vec{\text{X}}}^{*} ({\text{t}})$$

The logarithmic spiral is defined by the constant *b*, and the value of l is chosen at random from [− 1, 1]. There is a 50% likelihood of using shrinking or the spiral technique to attack the prey successfully. Equation (6) defines the equation.

6 $${\vec{\text{X}}}({\text{t}} + 1) = \left\{ {\begin{array}{*{20}l} {{\vec{\text{X}}}^{*} ({\text{t}}) - {\vec{\text{A}}} \cdot {\vec{\text{D}}}} \hfill & {{\text{if}}\,p < 0.5} \hfill \\ {\overrightarrow {{{\text{D}}^{\prime } }} \cdot {\text{e}}^{{{\text{bl}}}} \cdot \cos (2\uppi {\text{l}}) + {\vec{\text{X}}}^{*} ({\text{t}})} \hfill & {{\text{if}}\,p \ge 0.5} \hfill \\ \end{array} } \right.$$

$p$ is a number between 0 and 1.

#### Search for prey

The last process to be discussed in the WOA process is how the target is searched. Humpback whales disperse and explore the search space at random to identify the target, as Eq. (3) describes. When the value of vector $A$ exceeds $1$ or falls below -1, it prompts the whales to spread out and perform a random search. The goal of this phase is to integrate exploratory abilities into the WOA. During this stage, the following location of the agents is determined at random, irrespective of the existing best solution’s value. Equations (7) and (8) explain the search process further.

7 $${\vec{\text{D}}} = \left| {{\vec{\text{C}}} \cdot {\vec{\text{X}}\text{r}} - {\vec{\text{X}}}} \right|$$

8 $${\vec{\text{X}}}({\text{t}} + 1) = {\vec{\text{X}}\text{r}} - {\vec{\text{A}}} \cdot {\vec{\text{D}}}$$

Vector ($X_{r}$) is randomly positioned and drawn from the selected population. WOA begins with randomly generated solutions and iteratively refines the optimal solution using either random search agents or the best-performing one. The technique is based on three key parameters: vector a, vector A, and vector *p*. Vector a, which diminishes through 2 to 0, is utilized to maintain a suitable equilibrium between exploration and exploitation. Vector *A* determines whether to use a random or the best search agent for position updating. Suppose vector *A* is more significant than one (1); a randomly generated search agent is used to effect an update on the position. However, the current best solution is used if it is less than one. This assists the WOA in maintaining the proper equilibrium between exploring and exploiting. *p* facilitates the search agents to vary between cyclic and spiral movement, increasing their adaptability.

#### Feature selection using WOA

The proposed modified WOA determines the best features using the whale optimization algorithm with input from the preprocessed data. The initial location of the whales (indicating feature selection or non-selection) is generated randomly as binary values. The algorithm then updates each whale’s position in each iteration, shrinking the search space with each iteration. The new position is determined by combining the best position found thus far with random numbers. If the new position has better fitness, it becomes the best. The algorithm then returns the indices of the selected features, considered the best feature. The returned indices are passed to the next step to aid the prediction process. Each whale represents a potential solution in the context of feature selection, with a binary vector encoding the presence or lack of features. The algorithm updates each whale’s location based on a linearly decreasing search space coefficient and the best position over a predetermined number of iterations (10, 20, 30, 40, 50 to 100) and agents (10, 20, 30, 40, 50 to 100).

Ten agents are paired against ten iterations for the WOA. This is repeated in additions of 10 (for agents and iterations). By training a Logistic Regression classifier penalized by an L1 regularization on the chosen feature sets, the fitness function is utilized to compute the performance of the provided subset of features. Each iteration updates the whale position. If the new position improves fitness, the position and fitness are updated accordingly. The maximized prediction performance on the validation with a minimized number of features is considered the optimal feature for that iteration. The best position, therefore, reflects the ideal subset of features so far as the algorithm has run through the required number of iterations. The Scaled training data and its target, the number of whales and iterations are the input factors used by WOA for feature selection. The entire process is captured in Algorithm 1.

**Algorithm 1:**
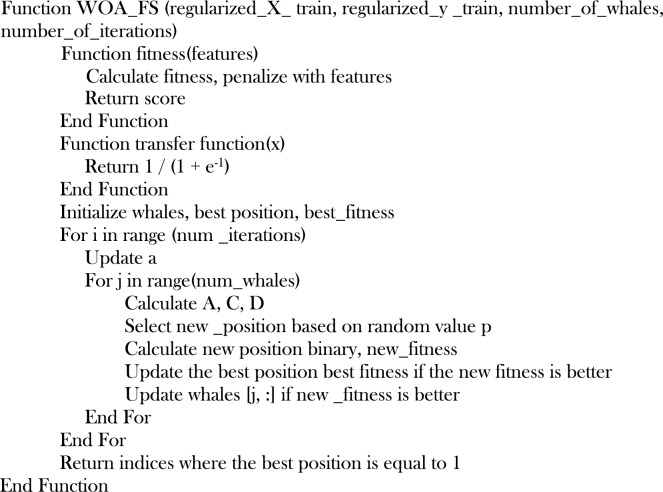
.

##### Fitness function

Our study presents a novel approach to the fitness function in the whale optimization algorithm (WOA). Central to this method is integrating a logistic regression model with L1 regularization, a choice motivated by the model’s inherent capacity for feature selection and sparsity^52^.

The logistic regression model, known for its effectiveness in binary classification problems, is enhanced with L1 regularization. This regularization technique introduces a penalty term equivalent to the absolute value of the magnitude of the coefficients. The primary advantage of incorporating L1 regularization is its tendency to produce sparse solutions, inherently performing feature selection by driving the coefficients of less significant features to zero^54,55^. This aspect is particularly beneficial in our study, where model simplicity and interpretability are paramount.

In evaluating the fitness of the WOA, we adopt a cross-validation with five folds. This partitions the data into five subsets, iteratively using one subset for validation and the rest for training. Such a technique strengthens the model’s validation on different data samples and mitigates the risk of overfitting, leading to a more reliable performance evaluation^56^.

Also, while L1 regularization naturally promotes feature sparsity, our approach further penalizes the fitness score based on the number of features the logistic regression model selects. This penalty is designed to encourage the selection of the most relevant features and enhance interpretability.

With its unique integration of logistic regression with L1 regularization, k-fold cross-validation, and feature selection penalty, our proposed fitness function is a robust tool in the whale optimization algorithm. It adheres to the principles of parsimony and generalizability and aligns to achieve high predictive accuracy while maintaining model simplicity. This approach has significant implications for applications in various domains, particularly those involving high-dimensional datasets where feature selection is critical in model performance.

##### Transfer function

In the research, a sigmoid transfer is defined for use in WOA. The sigmoid function is mathematically depicted as Eq. (9).

9 $$f\left( {\text{x}} \right) = \frac{1}{{1 + {\text{e}}^{ - 1} }}$$

The sigmoid function can receive a numerical input in real numbers and subsequently act upon it by transforming input data into a finite range between 0 and 1. The sigmoid function converts the continuous output of the whale optimization algorithm into a binary format amenable to indicating the inclusion or exclusion of features. By employing a threshold value on the sigmoid, established at 0.5, we can ascertain the appropriateness of incorporating a feature, denoted as ‘1’, or removing it as ‘0’.

##### Optimal features selected

WOA is a metaheuristic algorithm with stochastic characteristics; hence, it generates marginally distinct feature sets for every dataset in ten (10) different runs. WOA was executed ten times for every dataset. The selected frequency for each feature was determined by tallying the occurrences across ten separate runs indicating the prominence of each feature. The features that exhibit the highest occurrence among the various iterations are considered potential optimal features due to their higher consistency and relevance. To determine the final set of optimal features, a threshold value of 80% was established. This threshold ensures that only those features that appear in at least 80% of the experimental runs (a minimum of 8 out of 10 runs) will be selected. The optimal features chosen are subjected to individual evaluation by executing the classifiers. The performance of these features is documented for further analysis. The findings offer valuable insight into the effects of feature selection on model performance. Using the methodology above, the experiment endeavours to alleviate the stochastic nature inherent in the whale optimization algorithm (WOA) and produce a resilient collection of optimal features for diagnosing heart disease risk. Table 2 outlines the various datasets and the optimal features identified and selected.

**Table 2:** Optimal features selected.

#	Dataset	Total features	No. of features selected	Optimal features selected
1	Combined	13	9	Age, cp, trestbps, fbs, thalach, exang, oldpeak, ca, thal
2	Z-AlizadehSani dataset	53	34	Age, sex, BMI, DM, HTN, current smoker, FH, CVA, DLP, lung rales, systolic murmur, typical chest pain, dyspnea, function class, atypical, nonanginal, exertional CP, Q wave, St elevation, St depression, tinversion, LVH, poor R progression, FBS, TG, LDL, HDL, ESR, HB, Na, Neut, PLT, EF-TTE, VHD
3	Framingham heart study	15	9	Male, age, cigsperday, prevalentstroke, prevalenthyp, totchol, sysbp, heartrate, glucose
4	SA heart dataset	9	7	Sbp, tobacco, ldl, adiposity, famhist, typea, age
5	Cleveland UCI	13	9	Age, cp, trestbps, fbs, thalach, exang, oldpeak, ca, thal

##### Visualization after feature selection

**Figure 9:**
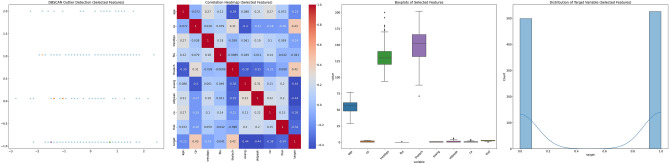
Combined dataset visualization after feature selection.

**Figure 10:**
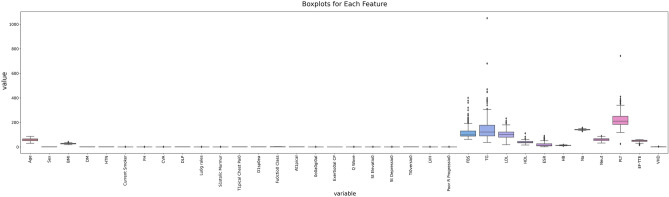
DBAZ-AlizadehSani dataset BoxPlot visualization after feature selection.

**Figure 11:**
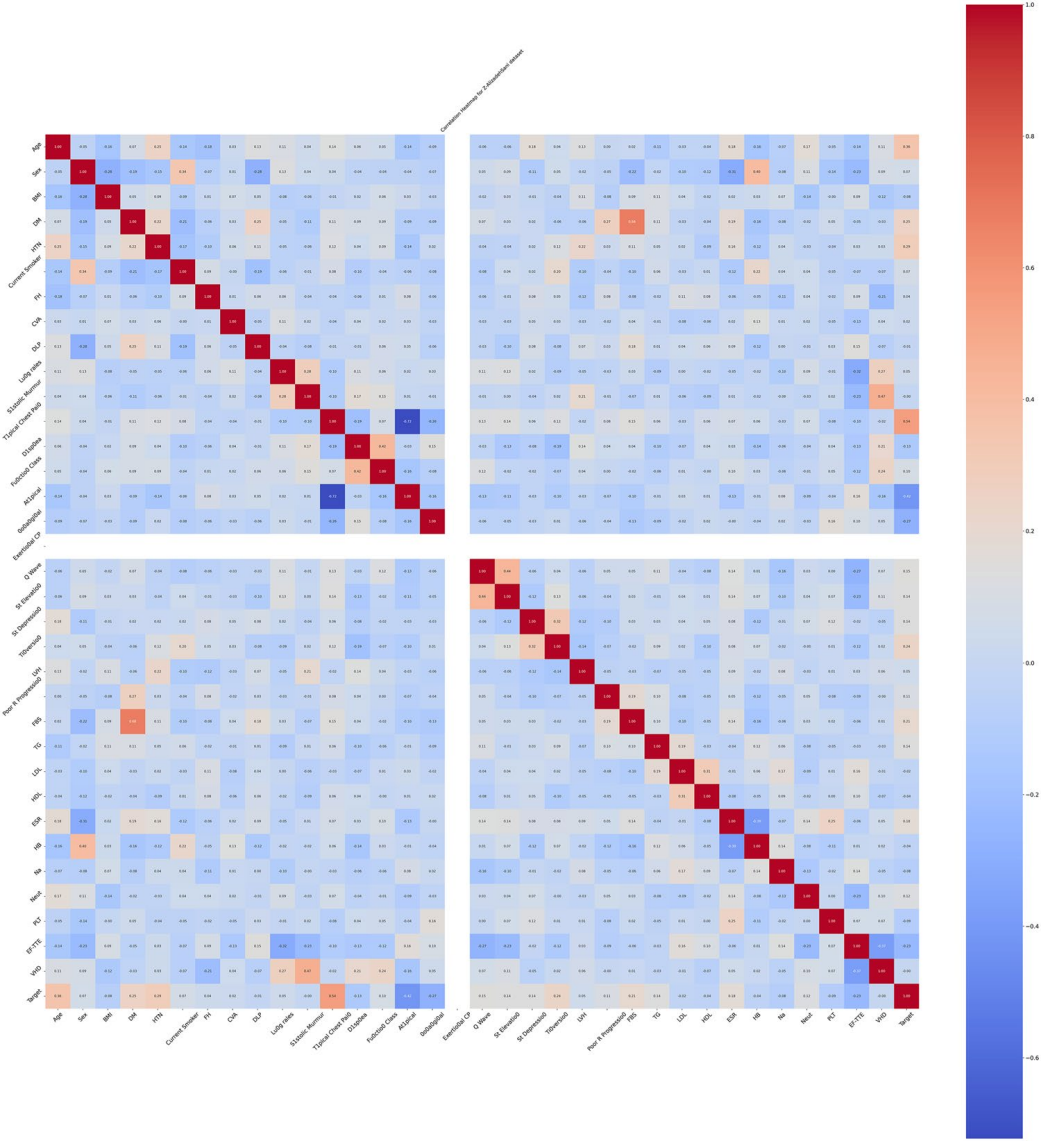
DBAZ-AlizadehSani dataset BoxPlot visualization after feature selection.

**Figure 12:**
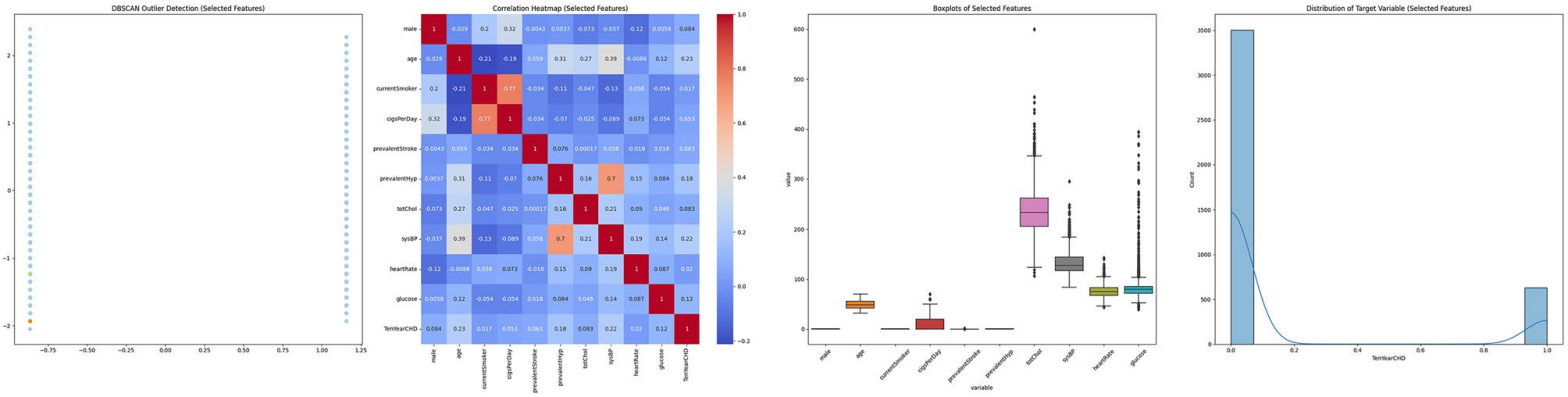
Framingham dataset visualization after feature selection.

**Figure 13:**
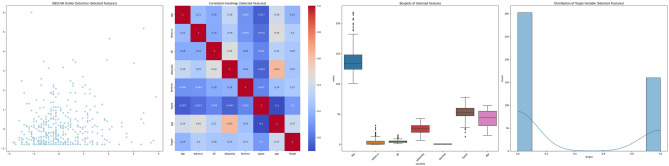
South African heart dataset visualization after feature selection.

**Figure 14:**
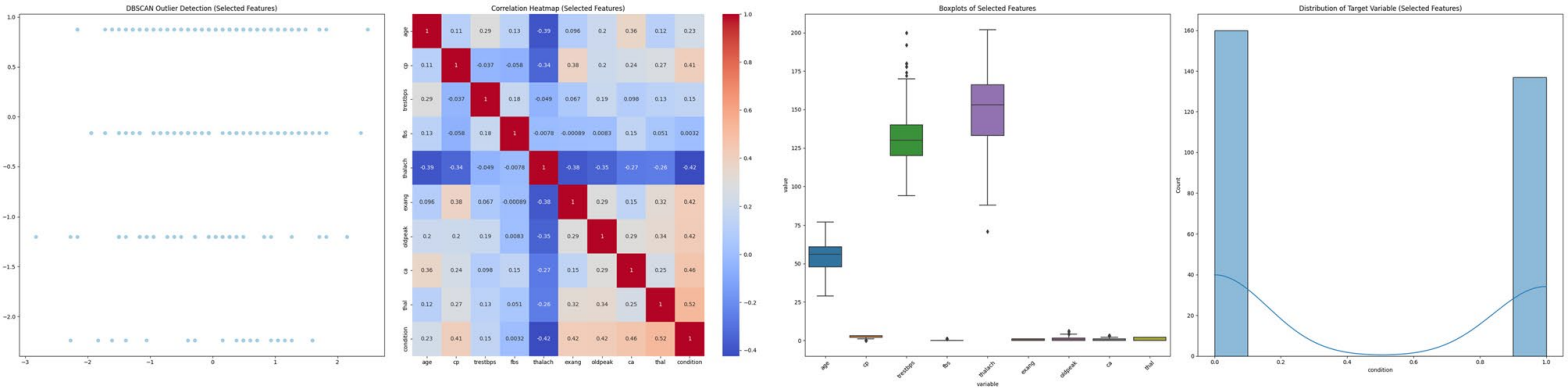
Cleveland UCI dataset visualization after feature selection.

The DBSCAN Outlier Detection scatter plots across the datasets illustrate the DBSCAN algorithm’s ability to identify clusters and outliers. The clusters appear more distinct in the selected features compared to the broader spread seen in the full feature set. This suggests that feature selection has potentially removed noisy variables, allowing for more apparent patterns to emerge. The Correlation Heatmaps provide a detailed view of the interdependencies between features. After feature selection, the heatmaps are generally less cluttered, with fewer variables exhibiting strong correlations. This reduction in multicollinearity can benefit many machine learning models, as it tends to enhance model interpretability and performance. The boxplots also highlight the distribution and variance of each feature within the datasets. Post-feature selection, the plots are fewer but more focused, often showing a reduced number of extreme outliers. This indicates that feature selection has likely discarded features with extreme values that could skew the model’s learning process. The shapes of the target distributions are generally consistent before and after feature selection, signifying that the selected features maintain the original structure of the target variable. However, in some cases, the distribution appears more balanced post-feature selection, which may positively influence model performance, especially in classification tasks. In all, the visualization suggests that feature selection has streamlined the datasets, potentially improving the efficiency and efficacy of subsequent analyses. By focusing on the most informative features, we expect that the chosen subsets will provide clearer, more relevant insights and facilitate the development of more robust predictive models. Figures 9, 10, 11, 12, 13 and 14 present a visual representation of the various features after performing feature selection.

### Training and testing models

After selecting the optimal features, the data is passed to the prediction model for training and testing. The models used are Support Vector Machine, Decision Tree, Random Forest, Multi-Layer Perceptron, Recurrent Neural Network, Adaptive Boosting, Long Short-Term Memory, Extreme Gradient Boosting, K-Nearest Neighbors, and Naïve Bayes classifiers. The choice of prediction models used ranges from classical ML classifiers, ensemble classifiers, and deep learning classifiers to get a broader perspective on performance.

#### Model evaluation metrics

The models are evaluated using accuracy, precision, recall, AUC and F1 score. The evaluation metrics utilized and the reasons for the choice are discussed next.

##### Accuracy

Accuracy measures the frequency with which a model accurately predicts outcomes. This metric provides a simple and intuitive understanding of how well the model performs regarding correct classifications^57,58^ making it a valuable choice for this work. The formula for calculating accuracy is provided in Eq. (10).

10 $${\text{Accurancy}} = \frac{{({\text{TP}} + {\text{TN}})}}{{{\text{TP}} + {\text{TN}} + {\text{FP}} + {\text{FN}}}}$$

##### Precision

Precision primarily focuses on minimizing false positives to avoid unnecessary stress and medical interventions for patients^59^. High precision indicates that the model accurately identifies heart disease cases, facilitating informed clinical decision-making^60^. This is crucial in building trust in the model’s diagnostic capabilities, hence its usage in our work. The precision formula is listed as Eq. (11).

11 $${\text{precision}} = \frac{{{\text{TP}}}}{{{\text{TP}} + {\text{FP}}}}$$

##### Recall

Recall, also called sensitivity, is the ratio of correctly classified positive samples to all samples assigned to the positive class^11^. It shows the percentage of positive samples that are correctly classified. Given that a high recall is achieved by missing as few positive instances as possible, this metric is also thought to be among the most crucial for medical research. It is depicted by Eq. (12).

12 $${\text{Recall}} = \frac{{{\text{TP}}}}{{{\text{TP}} + {\text{FN}}}}$$

##### AUC

The Area Under the Curve (AUC) of the Receiver Operating Characteristic (ROC) is a metric that measures the model’s ability to distinguish between the negative and positive classes^61^. It is calculated based on the ROC curve, which plots the True Positive Rate (TPR) against the False Positive Rate (FPR) at various thresholds^62^. Computed using Eq. (13), a higher AUC value indicates overall model performance, signifying a high rate of correctly identified positive cases (sensitivity) and a low rate of false positives.

13 $${\text{AUC}} = \frac{{\left( {1 + {\text{TRP}} - {\text{FPR}}} \right)}}{2}$$

##### F1 score

Simple accuracy can be misleading as a model could inaccurately appear highly accurate by predominantly predicting the majority class^63,64^. To avoid this issue, F1 score is used. F1 score is the balance mean precision and recall^65^ and defined by the Eq. (14).

14 $${\text{F1}} = 2 \cdot \frac{{\left( {{\text{Precision}}*{\text{Recall}}} \right)}}{{\left( {{\text{Precision}} + {\text{Recall}}} \right)}}$$

#### Model hyperparameters

Table 3 lists the optimal hyperparameters for each of the ten models utilized in this work.

**Table 3:** Model hyperparameters.

#	Classifier	Hyperparameters
1	Adaptive boosting	n_estimators = 50, max_depth = 1, learning_rate = 1, algorithm = SAMME.R
2	Decision tree	max_depth = 3, random_stae = 42
3	K-nearest neighbor	n_neighbors = 3, weights = uniform, algorithm = auto,leaf_size = 30, p = 2, metric = default
4	Multi-layer perceptron	Hidden_layer_sizes = 100, activation = relu, solver = adam, learning_rate = constant
5	Long short-term memory	batch_size = 32, epochs = 75, learning_rate = 0.001, LSTM Units = 50, LSTM layers = 7, optimizer = adam, activation = sigmoid
6	Naïve Bayes	Guassian Naïve Bayes with var_smoothing of 1e-9
7	Random forest	n_estimators = 100, max_depth = 5
8	Recurrent neural network	Number of units in RNN = 32, activation function = sigmoid, optimizer = adam, loss function = binary_crossentropy, epochs = 20, batch_size = 32
9	eXtreme gradient boosting	Objective = binary:hinge, random stae = 42, n_estimators = 100, learning_rate = 0.3, max_depth = 6, subsample = 1, colsample_bytree = 1, min_child_weight = 1
10	Support vector machine	Kernel = linear, class_weight = balanced. All other hyperparameters are left in default

### Experimental setup

In this study, we conduct experiments on a Dell Latitude 5430 laptop with a 12th Gen Intel(R) Core (TM) i5 1245U CPU, 16 GB of RAM, and 12 logical processors running Windows 10.

### Ethical and informed consent

All ethical and informed consent for data use has been taken care of by the data providers.

## Results and discussions

This section discusses the results obtained from the baseline studies and the experiments conducted with feature selection (WOA).

### Baseline experiments without feature selection

The baseline experiments in this subsection serve as a critical foundation, providing valuable insights and setting a benchmark for the subsequent evaluation of feature selection methods. The experimentation results are captured in Tables 4, 5 and 6.

**Table 4:** Combined and Z-AlizadehSani datasets without FS.

Dataset	Combined dataset					Z-AlizadehSani dataset				
Classifier	Acc. (%)	Prec. (%)	Rec. (%)	F1 (%)	AUC (%)	Acc. (%)	Prec. (%)	Rec. (%)	F1 (%)	AUC (%)
AdaBoost	89.27	86.96	93.46	90.09	89.08	80.33	84.78	88.64	86.67	73.73
DT	84.39	84.40	85.98	85.19	84.32	81.97	86.67	88.64	87.64	76.67
KNN	91.22	92.38	90.65	91.51	91.25	60.66	72.73	72.73	72.73	51.07
MLP	85.56	85.10	88.04	86.40	85.45	70.49	77.94	86.59	78.17	57.71
LSTM	88.88	89.43	88.63	88.77	88.63	74.75	55.88	59.22	55.08	59.22
NB	85.37	89.72	83.48	85.26	85.63	73.77	68.18	93.75	72.08	72.74
RF	94.20	90.61	87.56	91.95	92.39	88.52	99.77	86.47	92.63	92.73
RNN	83.32	83.69	83.09	83.18	83.09	72.13	36.07	50.00	41.90	50.00
XGB	95.70	95.10	97.59	96.13	95.82	84.43	94.32	85.54	89.71	83.00
SVM	85.37	91.59	82.35	86.73	85.94	83.61	88.64	88.64	88.64	79.61

**Table 5:** Framingham and cleveland UCI datasets without FS.

Dataset	Framingham dataset					Cleveland-UCI				
Classifier	Acc. (%)	Prec. (%)	Rec. (%)	F1 (%)	AUC (%)	Acc. (%)	Prec. (%)	Rec. (%)	F1 (%)	AUC (%)
AdaBoost	85.13	57.89	8.73	15.17	53.79	83.33	93.75	78.95	85.71	84.93
DT	84.52	33.33	1.59	3.03	50.51	68.33	88.00	57.89	69.84	72.13
KNN	82.47	32.73	14.29	19.89	54.50	60.00	81.82	47.37	60.00	64.59
MLP	84.75	49.30	5.87	10.21	52.40	81.17	94.69	74.74	83.16	83.50
LSTM	84.76	47.39	50.07	46.03	50.07	71.83	76.68	76.81	71.71	76.81
NB	82.95	18.25	37.70	57.49	62.13	83.33	76.32	96.67	83.03	83.33
RF	84.98	5.16	54.56	9.17	69.96	77.67	67.37	96.24	79.23	79.58
RNN	84.76	44.89	50.03	45.95	50.03	80.50	80.87	83.17	80.20	83.17
XGB	82.89	12.30	32.82	17.72	59.33	67.50	60.53	83.73	70.26	68.76
SVM	67.35	68.25	27.22	38.91	59.69	81.67	78.95	90.91	84.51	80.64

**Table 6:** SA heart datasets without FS.

Dataset	SA heart dataset				
Classifier	Acc. (%)	Prec. (%)	Rec. (%)	F1 (%)	AUC (%)
AdaBoost	69.89	58.33	43.75	50.00	63.68
DT	63.44	44.44	25.00	32.00	54.30
KNN	58.06	36.00	28.13	31.58	50.95
MLP	69.68	59.55	41.69	48.31	62.18
LSTM	64.49	56.07	52.11	50.70	54.16
NB	72.04	59.38	59.38	69.03	69.03
RF	70.97	41.25	61.56	49.36	67.66
RNN	68.71	65.78	59.51	57.86	59.51
XGB	65.59	45.31	50.24	47.63	61.43
SVM	0.741935	0.71875	0.6053	0.6571	0.7208

Key: Acc = Accuracy, Prec. = Precision, Rec. = Recall, F1 = F1 score.

### Experiments with WOA feature selection

This subsection discusses the findings from experiments with WOA feature selection.

#### Evaluating completion time for WOA feature selection

**Figure 15:**
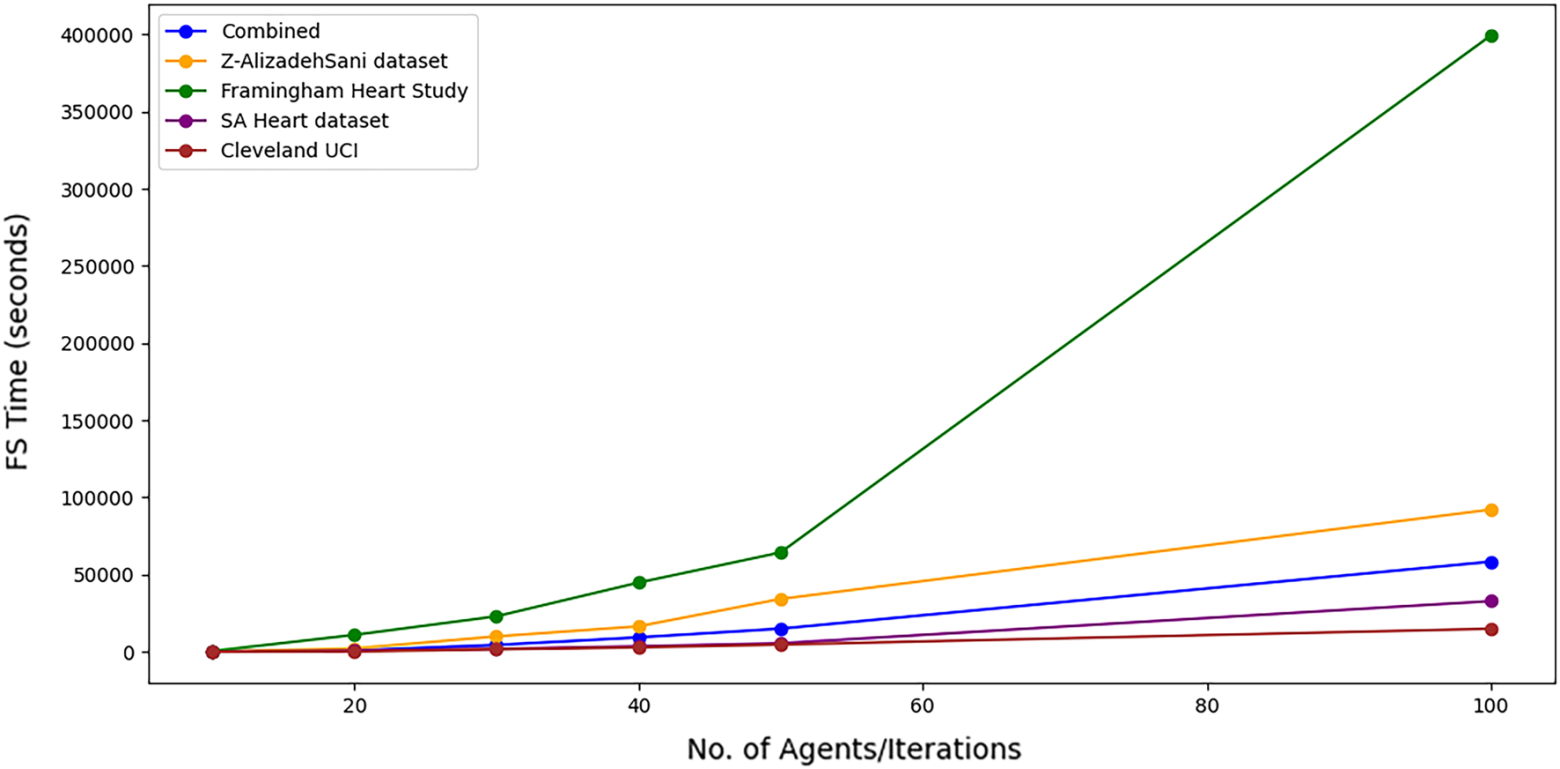
Feature selection time across datasets.

In this subsection, the study delves into the completion time when WOA feature selection is employed. We find that the duration varies across different datasets. Factors such as the number of agents, iterations, and features in the data directly influence the completion time; more factors result in a longer duration. Moreover, the observations in each dataset also impact the model completion time. For instance, despite the Z-AlizadehSani and Cleveland UCI datasets containing 303 records, the former requires more time for feature selection. Taking the Framingham dataset as an example, the highest time consumption occurred with this dataset. The completion times for 10, 20, 30, 40, 50, and 100 agents/iterations were 7.56 s, 182.26 s, 383.06 s, 747.03 s, 1072.46 s, and 6656.57 s, respectively. The SA Heart dataset, with times of 0.46 s, 11.80 s, 30.05 s, 58.56 s, 91.20 s, and 547.01 s, had a longer completion time than the Cleveland dataset, even though the Cleveland dataset possesses three additional features. This discrepancy could be due to the marginally more significant number of records in the Cleveland UCI dataset. In summary, the time required for WOA to complete the feature selection process increases with the number of features and records in each dataset. Figure 15 presents the findings about feature selection time using Whale Optimization.

#### Average estimation of evaluation metrics

**Figure 16:**
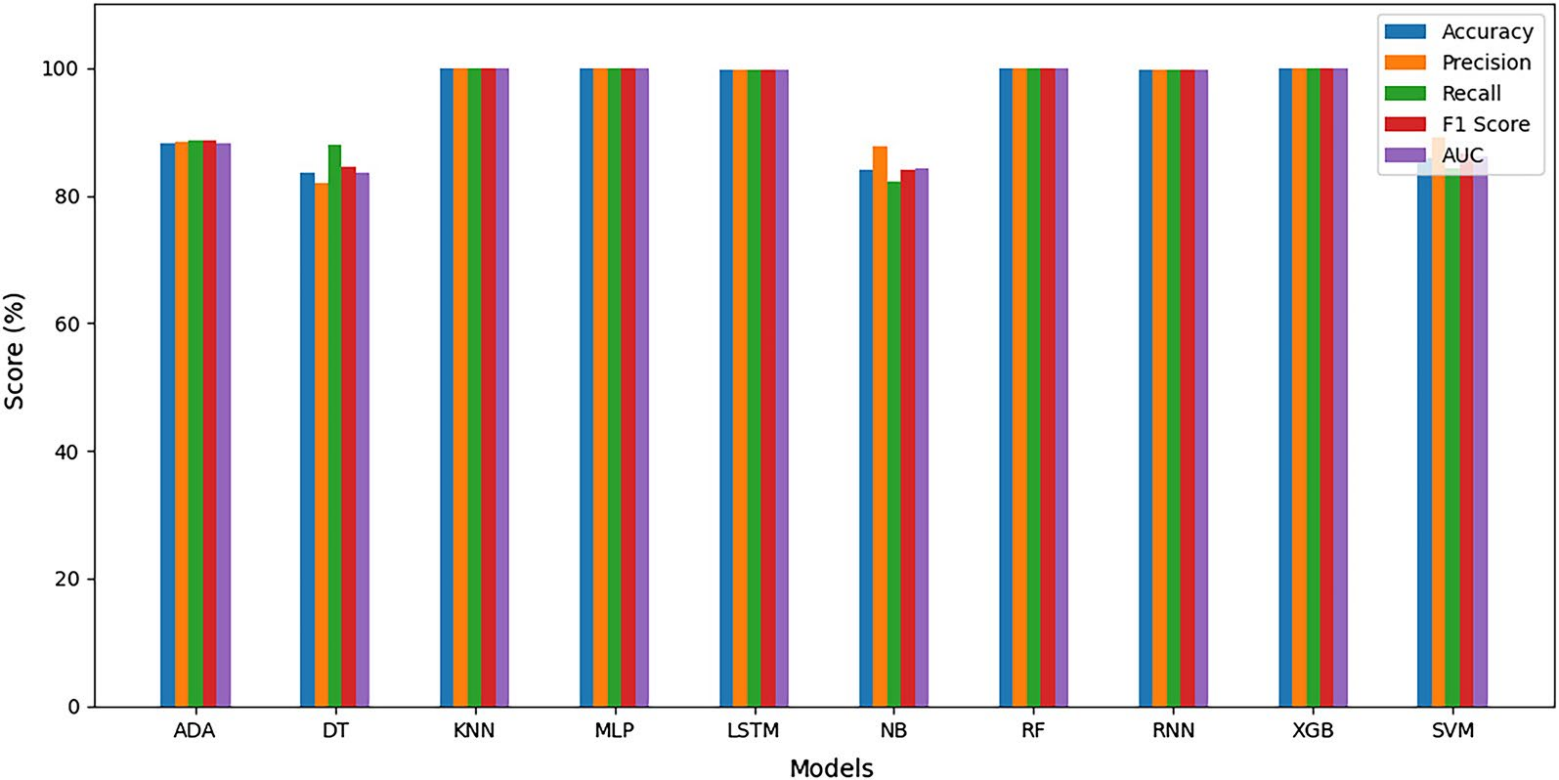
Average evaluation metrics for combined dataset.

**Figure 17:**
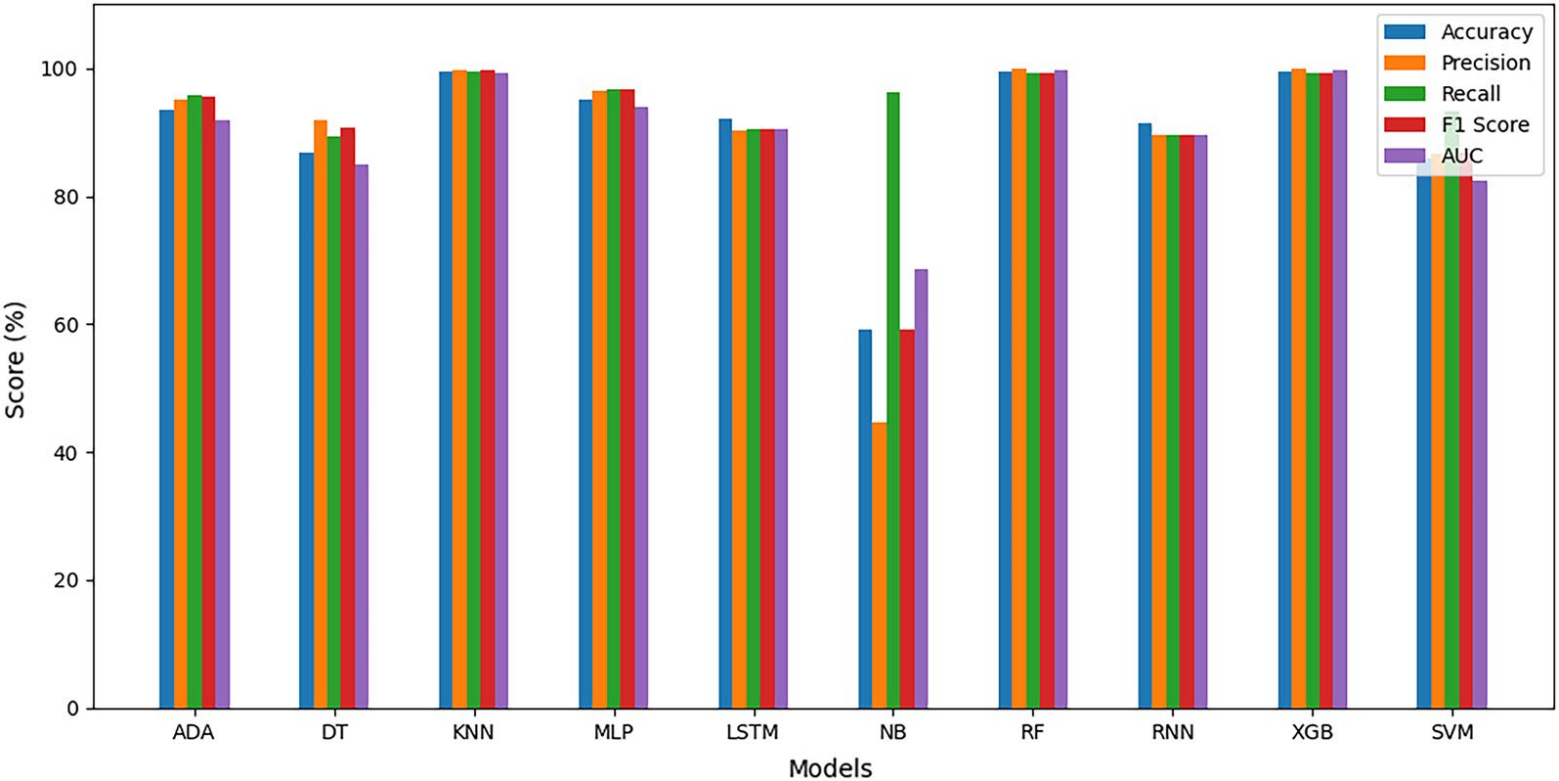
Average evaluation metrics for Z-AlizadehSani dataset.

**Figure 18:**
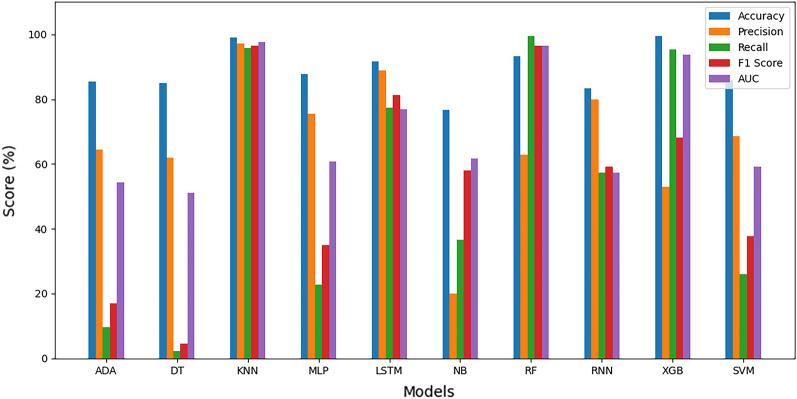
Average evaluation metrics for framingham dataset.

**Figure 19:**
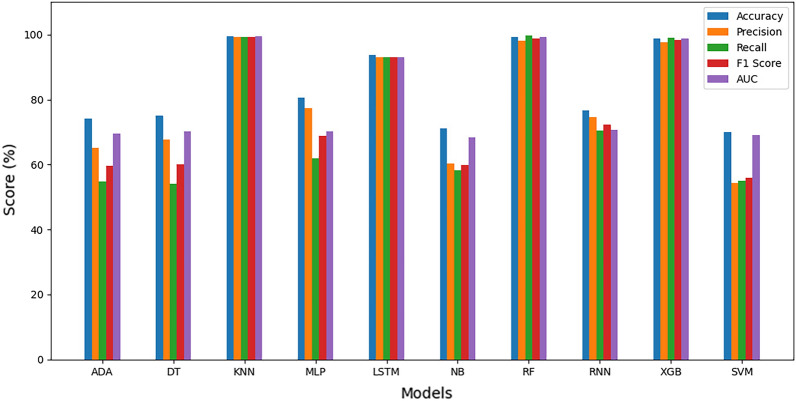
Average evaluation metrics for SA heart dataset.

**Figure 20:**
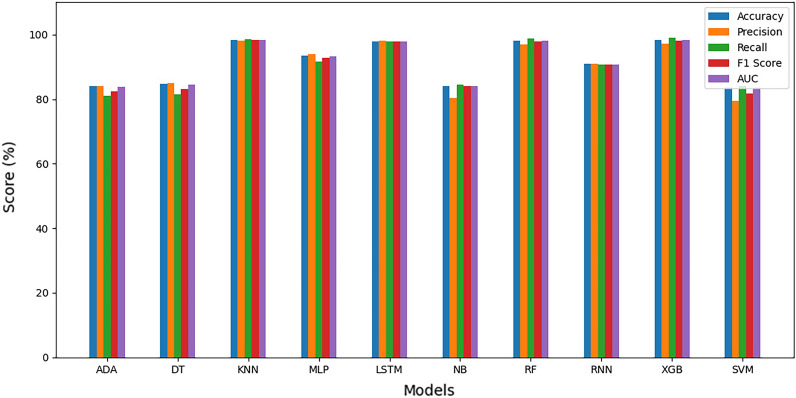
Average evaluation metrics for cleveland heart dataset.

To obtain a general overview of the evaluation metrics, an average is computed for each metric after ten (10) runs for each of the five (5) datasets and all ten (10) models. The results show a general improvement across all metrics and datasets. The average metrics are elaborated in Figs. 16, 17, 18, 19, and 20, depicted per dataset.

#### Evaluating metrics using optimal features

The KNN model performs best across all the metrics and for all the datasets. The results significantly improved over the same experiments on all datasets without WOA feature selection. In some instances, KNN with WOA records 100% across metrics (combined dataset), indicating the influence of WOA on a model’s performance. XGB models perform very well across all the metrics on all datasets except the Framingham dataset. Though XGB performs lower on the Framingham dataset than on all the other datasets, the performance across all the valuation metrics is significantly improved over values recorded without WOA feature selection on the Framingham dataset. In essence, the model has improved due to the feature selection just lower than recorded for other datasets when feature selection is employed. The RF classifier also performs well on all the datasets except the Framingham dataset, which performs well on all the metrics apart from the lowest precision (4.78%). The LSTM model records the highest, 99.80%, on all the metrics except the AUC, which recorded 99.81%. The best performance is recorded on the combined dataset for all the models. Similar performance improvement is seen across all the other datasets for the deep learning models. Generally, the ensemble models (RF, XGB and AdaBoost recorded consistent and improved performance across all the datasets on the evaluation metrics than all but KNN for the classical ML models. The deep learning models perform comparatively well, especially on larger datasets. It was also observed that though the SA heart dataset has a relatively small number of features (9 features), WOA selected seven, subsequently improving the model metrics across all the models. This shows that even if there are not many features, WOA can still select the optimal features if they exist. The experiments consistently improve the evaluation metrics when the results from the model with feature selection are compared to those without. The evaluation metrics are captured in Tables 7, 8 and 9.

**Table 7:** Model performance on combined and Z-AlizadehSani datasets using optimal features.

Dataset	Combined dataset					Z-AlizadehSani dataset				
Classifier	Acc. (%)	Prec. (%)	Rec. (%)	F1 (%)	AUC (%)	Acc. (%)	Prec. (%)	Rec. (%)	F1 (%)	AUC (%)
SVM	82.05	87.83	79.38	83.39	82.47	85.48	84.72	94.33	94.33	89.27
NB	81.85	84.79	80.80	81.81	81.94	60.40	46.76	95.28	95.28	60.24
RF	91.32	94.49	89.23	91.78	91.52	92.74	96.30	93.69	93.69	94.98
XGB	100.00	100.00	100.00	100.00	100.00	98.35	99.07	98.62	98.62	98.85
AdaBoost	87.02	86.59	88.40	87.49	86.99	92.74	94.50	95.37	95.37	94.93
DT	82.83	81.70	85.74	83.67	82.75	85.48	93.88	85.19	85.19	89.32
KNN	99.80	99.62	100.00	99.81	99.80	99.01	100.00	98.61	98.61	99.30
RNN	98.93	98.92	98.93	98.93	98.93	94.72	93.82	93.21	93.21	93.51
MLP	99.41	99.24	99.62	99.43	99.41	97.03	98.59	97.22	97.22	97.90
LSTM	99.80	99.80	99.81	99.80	99.81	94.72	92.90	94.58	94.58	93.68

**Table 8:** Model performance on framingham and cleveland datasets using optimal features.

Dataset	Framingham dataset					Cleveland-UCI				
Classifier	Acc. (%)	Prec. (%)	Rec. (%)	F1 (%)	AUC (%)	Acc. (%)	Prec. (%)	Rec. (%)	F1 (%)	AUC (%)
SVM	65.71	83.16	83.16	83.16	83.16	83.16	84.65	87.58	87.58	86.09
NB	83.79	83.50	83.50	83.50	83.50	83.50	85.83	86.51	86.51	84.59
RF	85.53	90.24	90.24	90.24	90.24	90.24	93.31	88.76	88.76	90.98
XGB	90.08	96.97	96.97	96.97	96.97	96.97	97.83	97.07	97.07	97.45
AdaBoost	85.63	83.16	83.16	83.16	83.16	83.16	89.30	90.35	90.35	89.82
DT	84.93	85.52	85.52	85.52	85.52	85.52	85.77	88.98	88.98	87.34
KNN	99.44	98.32	98.32	98.32	98.32	98.32	99.41	98.82	98.82	99.11
RNN	85.97	90.91	90.91	90.91	90.91	90.91	91.95	91.97	91.97	91.96
MLP	87.15	91.92	91.92	91.92	91.92	91.92	95.12	95.87	95.87	95.49
LSTM	92.52	98.99	98.99	98.99	98.99	98.99	94.68	94.76	94.76	94.72

**Table 9:** Model performance on SA heart.

Dataset	SA heart disease				
Classifier	Acc. (%)	Prec. (%)	Rec. (%)	F1 (%)	AUC (%)
SVM	68.83	73.13	53.67	53.67	61.90
NB	71.65	61.88	58.58	58.58	69.08
RF	80.30	57.50	80.00	80.00	66.91
XGB	97.40	95.63	96.84	96.84	96.23
AdaBoost	76.41	68.35	59.38	59.38	63.55
DT	74.68	68.07	50.63	50.63	58.06
KNN	99.57	100.00	98.75	98.75	99.37
RNN	76.19	73.84	72.24	72.24	72.85
MLP	85.06	85.83	68.13	68.13	75.96
LSTM	82.90	81.00	82.22	82.22	81.50

Key: Acc = Accuracy, Prec. = Precision, Rec. = Recall, F1 = F1 score.

### Comparative studies

This section performs a comparative study with other related works, comparing the metrics used in the study with values from the metrics of the optimal features used in this work. Works used are the most recent studies in the domain that utilize the same datasets. Table 10 outlines recent studies with the same five datasets used in this work. We compared the features selected by the respective algorithms and their metrics reported and compared it with results from the optimal features in Tables 6, 7 and 8. The results when WOA is used show significant improvement.

**Table 10:** Comparative studies with recent works.

#	Author	#Features	Dataset	FS method	Metric
1	Wadhawan and Maini^66^	9	Combined	Optimal feature subset selection algorithm (OFSSA)	Accuracy: 99.666%, Precision: 99.832%, Recall/sensitivity: 99.832%, F1: 99.832%, AUC: N/A
2	Ghosh et al.^18^	10	Combined	Filter (relief and lasso)	Accuracy 99.05%
3	Kolukisa and Bakir-Gungor^67^	25	Z-AlizadehSani	Hybrid method (exhaustive and probabilistic ensemble)	Accuracy: 91.14%, Precision: 93.53%, Recall/sensitivity: 94.41%, F1: 93.7% 99.832%, AUC: 94.1%
4	Fajri et al.^68^	24	Z-AlizadehSani	Hybrid (Bee swarm optimization algorithm combined with Q-learning for optimizing)	Accuracy: 90.1%, Precision: N/A, Recall/sensitivity: N/A, F1: N/A, AUC: 94.1%
5	Kolukisa and Bakir-Gungor^67^	9	Cleveland	Hybrid method (exhaustive and probabilistic ensemble)	Accuracy: 85.47%, Precision: 86.22%, Recall/sensitivity: 82.96%, F1: 83.9% 99.83%, AUC: 91.1%
6	El-Shafiey et al.^69^	7	Cleveland	Hybrid (Genetic algorithm and particle swarm optimization)	Accuracy: 91.40%, Precision: N/A, Recall/sensitivity: N/A, F1: N/A, AUC: N/A
7	Fajri et al.^68^	6	Cleveland	Hybrid (Bee swarm optimization method and Q-learning)	Accuracy: 84.4%, Precision: N/A, Recall/sensitivity: N/A, F1: N/A, AUC: 90.1%
8	Ay et al.^49^	N/A	Cleveland	Swarm algorithms (Cuckoo search (CS), Flower pollination algorithm (FPA), and Harris hawks optimization (HHO)	Accuracy: N/A, Precision: N/A, Recall/sensitivity: N/A, F1: 99.72%, AUC: 98.0%
9`	Budholiya et al.^70^	N/A	Cleveland	None	Accuracy: 91.80%, Precision: N/A, Recall/sensitivity: 85.71%, F1: 90.56%, AUC: N/A
10	Owusu et al.^71^	10	Cleveland	Filter (Extra tree classifie)	Accuracy: 99.75%, Precision: 94%, Recall/sensitivity: 87%, F1: 90%, AUC: 93.2%
11	Mienye and Su^72^	N/A	Framingham	None	Accuracy: 91%, Precision: 92%, Recall/sensitivity: 90%, F1: 91%, AUC: N/A
12	Rahim et al.^73^	6	Framingham	Filter (feature importance)	Accuracy: 99.1%, Precision: N/A, Recall/sensitivity: N/A, F1: N/A, AUC: N/A
13	Krishnani et al.^74^	N/A	Framingham	None	Accuracy: 96.71%, Precision: 98.94%, Recall/sensitivity: 94.4%, F1: 96.61%, AUC: N/A
14	Mahmoud et al.^75^	N/A	Framingham	Embedded method	Accuracy: 85.05%, Precision: N/A, Recall/sensitivity: N/A, F1: 91.90%, AUC: N/A
15	Nalluri et al.^76^	N/A	Framingham	None	Accuracy: 85.86%, Precision: N/A, Recall/sensitivity: N/A, F1: N/A, AUC: N/A
16	Anuradha and David^77^	6	SA heart	Filter	Accuracy: 78.49%, Precision: 78%, Recall/sensitivity: 91%, F1: 84%, AUC: N/A
17	Khdair and Dasari^78^	N/A	SA heart	None	Accuracy: 78.1%, Precision: 78.4%, Recall/sensitivity: 77.6%, F1: 78%, AUC: N/A
18	Gokulnath and Shantharajah^79^	7	Cleveland UCI	Wrapper (genetic algorithm)	Accuracy: 88.34%, Precision: N/A, Recall/sensitivity: N/A, F1: N/A, AUC: N/A
19	Cenitta et al.^80^	13	Combined	Swarm (squirrel search feature selection algorithm)	Accuracy: 98.38%, Sensitivity: 98.66%, Specificity: 98.10%, F1: 98.32%
20	Zhang et al.^23^	12	Cleveland UCI	Embedded (LinearSVC Algorithm)	Accuracy: 98.56%, Precision: 97.84%, Recall: 99.35% Specificity: N/A, F1: N/A
21	Haq et al.^10^	6	Cleveland UCI	Wrapper (sequential backward selection)	Accuracy: 90.00%, Precision: N/A, Recall: N/A, Specificity: N/A, F1: N/A
22	Proposed model	9	Combined	Swarm (whale optimization algorithm)	Accuracy: 100.00%, Precision: 100.00%, Recall/sensitivity: 100.00%, F1: 100.00%, AUC: 100.00%
23	Proposed model	35	Z-AlizadehSani	Swarm (whale optimization algorithm)	Accuracy: 99.01%, Precision: 100.00%, Recall/sensitivity: 98.61%, F1: 98.61%, AUC: 99.30%
24	Proposed model	9	Framingham	Swarm (whale optimization algorithm)	Accuracy: 99.44%, Precision: 98.32%, Recall/sensitivity: 98.32%, %, F1: 98.32%, %, AUC: 98.32%
25	Proposed model	7	SA heart	Swarm (whale optimization algorithm)	Accuracy: 98.32%, Precision: 99.41%, Recall/sensitivity: 98.82%, F1: 98.82%, AUC: 99.11%
26	Proposed model	9	Cleveland UCI	Swarm (whale optimization algorithm)	Accuracy: 99.57%, Precision: 100%, Recall/sensitivity: 98.75%, F1: 98.75%, AUC: 99.37%

The results show superior metrics recorded when WOA feature selection is used and the impact of WOA on the model performance. It is worth noting that this work measures five evaluation metrics over five different datasets using ten models, providing an exhaustive list of metrics capable of judging the performance reported for every model.

Table 10 lists the most recent works in the HD domain, with the last five being the results obtained from this study on the optimal features.

### Limitations of the study

The study has data limitations as image heart disease datasets are out of the scope of this work. The experiments in this study are not performed for more than 100 agents and 100 iterations for the whale optimization algorithm due to computational constraints. Finally, the work is limited to only the heart disease domain.

## Conclusion

The original WOA is designed for continuous optimization. In this work, however, we implement the solution space into a binary form, necessitating the transformation of the output from continuous to binary using a sigmoid transfer function. A thresholding process is then used to convert the values to a binary format. We specify a fitness function which involves training a logistic regression model using the selected features and evaluating its performance through cross-validation. Since the aim is feature selection, the logistic regression model is penalized using an L1 (Lasso) to produce more sparse models, where a subset of coefficients becomes exactly zero, technically performing feature selection. We then introduce a penalty term to discourage the selection of more features. The whale optimization algorithm for feature selection shows improved heart disease risk prediction evaluation metrics. With fewer yet relevant features, models significantly improved across all five metrics. This contributes to the ongoing efforts to enhance the effectiveness of cardiovascular health risk, providing valuable insights for future studies in this field.

Future works can consider using WOA on image datasets for feature extraction. How WOA compares to other swarm intelligence algorithms in the heart disease domain is an area worth researching. Extending the research to more than one dataset in domains other than Cardiovascular health is a useful area for future studies. In addition, future studies can consider more hyperparameter tuning of all the models to improve the results obtained.

## Data Availability

Combined dataset https://www.kaggle.com/datasets/johnsmith88/heart-disease-dataset, Z-AlizadehSani dataset https://www.kaggle.com/datasets/tanyachi99/zalizadeh-sani-dataset-2csv, Framingham dataset https://www.kaggle.com/datasets/captainozlem/framingham-chd-preprocessed-data, South African Heart dataset https://hastie.su.domains/ElemStatLearn/datasets/SAheart.data, Cleveland UCI dataset https://www.kaggle.com/datasets/cherngs/heart-disease-cleveland-uci.

## Abbreviations

WOA: Whale optimization algorithm
FS: Feature selection
HD: Heart disease
ML: Machine learning
DL: Deep learning
KNN: K-nearest neighbor
RF: Random forest
DT: Decision tree
RNN: Recurrent neural network
LSTM: Long short-term memory
XGBoost: Extreme gradient boosting
NB: Naïve Bayes
SVM: Support vector machine
MLP: Multilayer perceptron
CSRS: Cuckoo search with the rough set
